# Misidentification of *Burkholderia pseudomallei*, China

**DOI:** 10.3201/eid2703.191769

**Published:** 2021-03

**Authors:** Bin Wu, Xinxin Tong, Haoyan He, Yinmei Yang, Huling Chen, Xiao Yang, Banglao Xu

**Affiliations:** Guangzhou First People’s Hospital, South China University of Technology, Guangzhou, China

**Keywords:** Burkholderia pseudomallei, misidentification, biochemical identification systems, China, bacteria, matrix-assisted laser desorption/ionization time-of-flight mass spectrometry

## Abstract

We report a case of melioidosis in China and offer a comparison of 5 commercial detection systems for *Burkholderia pseudomallei*. The organism was misidentified by the VITEK 2 Compact, Phoenix, VITEK mass spectrometry, and API 20NE systems but was eventually identified by the Bruker Biotyper system and 16S rRNA sequencing.

*Burkholderia pseudomallei* is the cause of melioidosis, a serious disease endemic to Southeast Asia and northern Australia (*1*). Because of the increase in international travel, the disease is now occurring in areas to which *B*. *pseudomallei* is not endemic. In these previously unaffected areas, laboratory staff might be unfamiliar with the organism or use identification systems that are not suitable for its detection, potentially leading to misidentification (*2*). We report the misidentification of *B*. *pseudomallei* by various commercial detection systems.

On May 15, 2019, a man 33 years of age in Guangxi Province, China, sought treatment for leg pain at a local hospital in Guangxi Province. Physicians diagnosed his condition as gout and prescribed oral febuxostat. However, the pain progressively worsened, and the patient began to have difficulty walking. On June 10 he was admitted to Guangzhou First People’s Hospital. Laboratory analysis of serum samples taken at admission showed moderate systemic inflammation with elevated levels of procalcitonin (0.296 ng/mL; reference value <0.05 ng/mL), C-reactive protein (61.7 mg/L; reference value <6.0 mg/L), erythrocyte sedimentation rate (120 mm/h; reference value <15 mm/h), leukocytes (13.87 × 10^9^ cells/L; reference value 1.1–3.2 × 10^9^ cells/L), and neutrophils (9.42 × 10^9^ cells/L; reference value: 1.8–6.3 × 10^9^ cells/L). His temperature fluctuated between 38.5°C and 39.8°C, peaking in the evening. Magnetic resonance imaging results suggested osteomyelitis. We conducted surgical debridement and collected pus from the lesion for microbiological analysis. We used the matrix-assisted laser desorption/ionization time-of-flight (MALDI-TOF) mass spectrometry VITEK 2 Compact system (bioMérieux, https://www.biomerieux.com) to identify the isolate as *Aeromonas sobria* with 93% probability. According to the VITEK 2 Compact system, the isolate was sensitive to amikacin, meropenem, imipenem, ceftazidime, ciprofloxacin, trimethoprim/sulfamethoxazole, and piperacillin/tazobactam but resistant to cefepime and aztreonam. We made a preliminary diagnosis of *Aeromonas* infection and treated the patient with piperacillin/tazobactam (500 mg, 4×/d) and levofloxacin (500 mg/d). However, we doubted the accuracy of this identification because *Aeromonas sobria* rarely causes extraintestinal disease (*3*). To examine this suspicion, we collected blood samples and incubated them in the Bact/ALERT 3D automated microbial detection system (bioMérieux). We cultured the samples on sheep blood and chocolate agar, revealing gram-negative rod-shaped bacteria (Figure; Appendix Figure 1). We then tested the samples with a variety of commercial detection systems. The VITEK 2 Compact system again identified the blood sample as *Aeromonas sobria* with 90% probability. However, the Bruker MALDI-TOF Biotyper system (Bruker Daltonics, https://www.bruker.com) identified the isolate as *B. pseudomallei* with an identification score of 2.18 (a score of >2.0 is considered an accurate identification). BD Phoenix M50 (Becton Dickinson, http://www.bd.com) identified it as *Alcaligenes faecalis* with 98% probability; VITEK MS (bioMérieux) identified it as *B. thailandensis* with an identification score of 2.23; API 20NE (bioMérieux) identified it as *Pseudomonas fluorescens* with 75.8% probability (Table).

**Figure:**
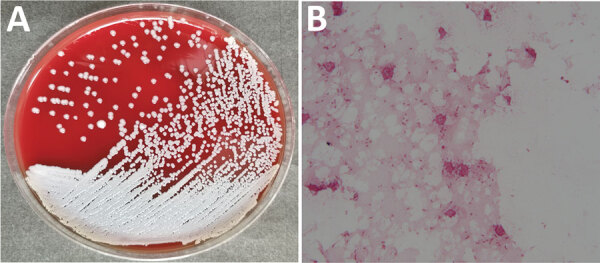
*Burkholderia pseudomallei* isolated from patient, China, 2019. A) Culture on sheep blood agar. B) Gram-stained smear. Original magnification ×1,000.

**Table:** Identification of *Burkholderia pseudomallei* by various detection systems, China, 2019

Detection method	Identification result	Characteristics
Vitek 2 Compact	*Aeromonas sobria*	90% probability
Phoenix	*Alcaligenes faecalis*	98% probability
Bruker Biotyper MS	*Burkholderia pseudomallei*	2.18 score*
Vitek MS	*Burkholderia thailandensis*	2.23 score*
API 20NE	*Pseudomonas fluorescens*	75.8% probability
16S rRNA	*Burkholderia pseudomallei*	GenBank accession no. CP040552.1

*An identification score >2.0 indicates an accurate identification.

To confirm the identity of the organism, we extracted DNA from blood cultures using a bacterial genomic DNA isolation kit (Sangon Biotech Co., Ltd, https://www.sangon.com). The 16S rRNA gene was amplified and sequenced by Sangon Biotech Co., Ltd. The isolate showed 100% identity and 100% coverage with a sequence of *B*. *pseudomallei* collected in India in 2019 (GenBank accession no. CP040552.1). On June 25, we diagnosed melioidosis in the patient. The patient recovered and was discharged after 14 days of the original piperacillin/tazobactam and levofloxacin treatment regimen. The global recommendations from the US Public Health Emergency Medical Countermeasures Enterprise suggest that physicians treat melioidosis with intravenous ceftazidime or meropenem, according to the severity of the disease; alternatively, physicians can prescribe oral trimethoprim/sulfamethoxazole or amoxicillin/clavulanic acid (*4*).

We conducted multilocus sequence typing as described previously (*5*). This isolate belongs to sequence type (ST) 550, corresponding with isolates previously documented in Vietnam in 2005 (*6*). The patient in this study had never been to Vietnam, but Guangxi Province borders that country. We constructed a phylogenetic tree with 1,000 bootstrap replicates using the unweighted pair group method with arithmetic averages in MEGA X software (https://www.megasoftware.net). This tree included isolates from other countries in Asia downloaded from PubMLST (https://pubmlst.org); the isolate in this study was most closely related to ST175 from Thailand (Appendix Figure 2) (*6*).

The accuracy of the identifications made by VITEK 2 (63%–81%), Phoenix (0%–28%), and API 20NE (37%–99%) systems varied substantially (*7*,*8*). Zakharova et al. found that commercially available biochemical identification systems commonly misidentified *B. pseudomallei* as *Chromobacterium violaceum* or *B. cepacia* complex (*9*). We found that although the isolate in this study was misidentified by multiple systems, most systems accurately identified the genus. MALDI-TOF mass spectrometry is a rapid, accurate, and highly reproducible technique for bacterial identification. Several studies have explored the potential of MALDI-TOF mass spectroscopy for the identification of *B. pseudomallei*. We prefer the Bruker Biotyper system, which is more accurate because the VITEK databases lack reference spectra for *B. pseudomallei* (*10*). In conclusion, scientists must be aware of the potential misidentification of *B. pseudomallei* by automated identification systems, especially those in regions to which *B. pseudomallei* is not endemic.

Appendix Additional information on misidentification of *Burkholderia pseudomallei*.

## References

[1] Chewapreecha C, Holden MT, Vehkala M, Välimäki N, Yang Z, Harris SR, et al. Global and regional dissemination and evolution of *Burkholderia pseudomallei.* Nat Microbiol. 2017;2:16263. 10.1038/nmicrobiol.2016.263 28112723 PMC5300093

[2] Kiratisin P, Santanirand P, Chantratita N, Kaewdaeng S. Accuracy of commercial systems for identification of *Burkholderia pseudomallei* versus *Burkholderia cepacia.* Diagn Microbiol Infect Dis. 2007;59:277–81. 10.1016/j.diagmicrobio.2007.06.013 17916419

[3] Kobayashi H, Seike S, Yamaguchi M, Ueda M, Takahashi E, Okamoto K, et al. *Aeromonas sobria* serine protease decreases epithelial barrier function in T84 cells and accelerates bacterial translocation across the T84 monolayer in vitro. PLoS One. 2019;14:e0221344. 10.1371/journal.pone.0221344 31419250 PMC6697317

[4] Lipsitz R, Garges S, Aurigemma R, Baccam P, Blaney DD, Cheng AC, et al. Workshop on treatment of and postexposure prophylaxis for *Burkholderia pseudomallei* and *B. mallei* Infection, 2010. Emerg Infect Dis. 2012;18:e2. 10.3201/eid1812.120638 23171644 PMC3557896

[5] Godoy D, Randle G, Simpson AJ, Aanensen DM, Pitt TL, Kinoshita R, et al. Multilocus sequence typing and evolutionary relationships among the causative agents of melioidosis and glanders, *Burkholderia pseudomallei* and *Burkholderia mallei.* J Clin Microbiol. 2003;41:2068–79. 10.1128/JCM.41.5.2068-2079.2003 12734250 PMC154742

[6] Kamthan A, Shaw T, Mukhopadhyay C, Kumar S. Molecular analysis of clinical *Burkholderia pseudomallei* isolates from southwestern coastal region of India, using multi-locus sequence typing. PLoS Negl Trop Dis. 2018;12:e0006915. 10.1371/journal.pntd.0006915 30418974 PMC6258418

[7] Zong Z, Wang X, Deng Y, Zhou T. Misidentification of *Burkholderia pseudomallei* as *Burkholderia cepacia* by the VITEK 2 system. J Med Microbiol. 2012;61:1483–4. 10.1099/jmm.0.041525-0 22820689

[8] Hoffmaster AR, AuCoin D, Baccam P, Baggett HC, Baird R, Bhengsri S, et al. Melioidosis diagnostic workshop, 2013. Emerg Infect Dis. 2015;21. 25626057 10.3201/eid2102.141045 PMC4313648

[9] Zakharova IB, Lopasteyskaya YA, Toporkov AV, Viktorov DV. Influence of biochemical features of *Burkholderia pseudomallei* strains on identification reliability by Vitek 2 System. J Glob Infect Dis. 2018;10:7–10. 10.4103/jgid.jgid_39_17 29563716 PMC5850765

[10] Lau SK, Sridhar S, Ho CC, Chow WN, Lee KC, Lam CW, et al. Laboratory diagnosis of melioidosis: past, present and future. Exp Biol Med (Maywood). 2015;240:742–51. 10.1177/1535370215583801 25908634 PMC4935216

