# Supplementary material for: Misidentification of Burkholderia pseudomallei, China
Source: Emerg Infect Dis. 2021 Mar;27(3):964–6. doi: 10.3201/eid2703.191769 (PMC7920660; doi:10.3201/eid2703.191769)
Supplement: Appendix — Additional information on misidentification of Burkholderia pseudomallei. [file 19-1769-Techapp-s1.pdf]

Article DOI: https://doi.org/10.3201/eid2703.191769

Misidentification of *Burkholderia pseudomallei*, China

Appendix

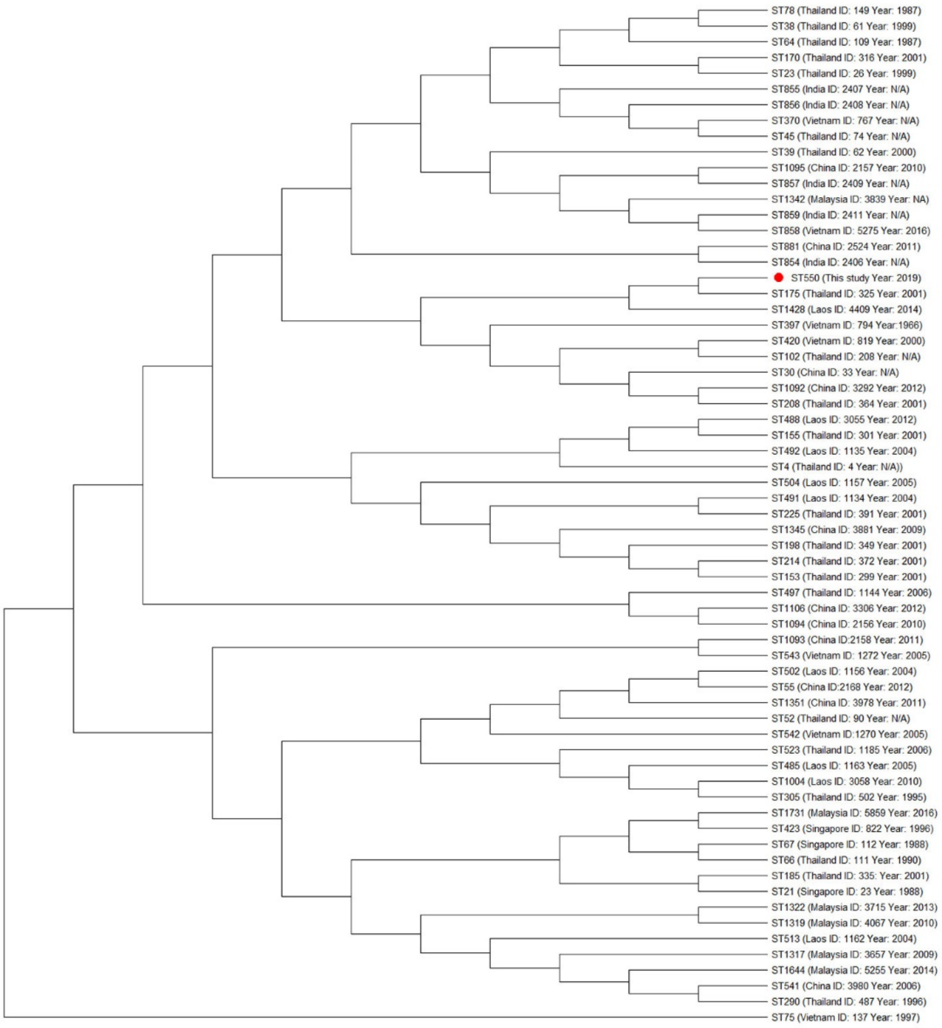

Appendix Figure. Phylogeny of 65 *Burkholderia pseudomallei* sequence type 550 isolates from countries in Asia. Red dot indicates isolate from this study, China, 2019. NA, no information available.
